# Pre-diagnostic circulating concentrations of fat-soluble vitamins and risk of glioma in three cohort studies

**DOI:** 10.1038/s41598-021-88485-0

**Published:** 2021-04-29

**Authors:** Yiyang Yue, Jordan H. Creed, David J. Cote, Meir J. Stampfer, Molin Wang, Øivind Midttun, Adrian McCann, Per Magne Ueland, Jeremy Furtado, Kathleen M. Egan, Stephanie A. Smith-Warner

**Affiliations:** 1 Department of Nutrition, Harvard T.H. Chan School of Public Health, Boston, MA USA; 2 Department of Biostatistics and Bioinformatics, H. Lee Moffitt Cancer Center, Tampa, FL USA; 3 Channing Division of Network Medicine, Brigham and Women’s Hospital, Boston, MA USA; 4 Department of Neurosurgery, Computational Neuroscience Outcomes Center, Brigham and Women’s Hospital, Boston, MA USA; 5 Department of Epidemiology, Harvard T.H. Chan School of Public Health, Boston, MA USA; 6 Department of Biostatistics, Harvard T.H. Chan School of Public Health, Boston, MA USA; 7 Bevital A/S, Bergen, Norway; 8 Department of Clinical Science, University of Bergen, 5021 Bergen, Norway; 9 Laboratory of Clinical Biochemistry, Haukeland University Hospital, Bergen, Norway; 10 Department of Cancer Epidemiology, H. Lee Moffitt Cancer Center, Tampa, FL USA

**Keywords:** Biomarkers, Nutrition, Cancer epidemiology

## Abstract

Few prospective studies have evaluated the relation between fat-soluble vitamins and glioma risk. Using three cohorts—UK Biobank (UKB), Nurses’ Health Study (NHS), and Health Professionals Follow-Up Study (HPFS), we investigated associations of pre-diagnostic concentrations of fat-soluble vitamins D, A, and E with incident glioma. In 346,785 participants (444 cases) in UKB, associations with vitamin D (25-hydroxyvitamin D [25(OH)D]) were evaluated by Cox proportional hazards regression. In NHS (52 cases, 104 controls) and HPFS (32 cases, 64 controls), associations with 25(OH)D, vitamin A (retinol), and vitamin E (α- and γ-tocopherol) were assessed using conditional logistic regression. Our results suggested plasma concentrations of 25(OH)D and retinol were not associated with glioma risk. Comparing the highest to lowest tertile, the multivariable hazard ratio (MVHR) for 25(OH)D was 0.87 (95% confidence interval [CI] 0.68–1.11) in UKB and the multivariable risk ratio (MVRR) was 0.97 (95% CI 0.51–1.85) in NHS and HPFS. In NHS and HPFS, the MVRR for the same comparison for retinol was 1.16 (95% CI 0.56–2.38). Nonsignificant associations were observed for α-tocopherol (MVRR_tertile3vs1_ = 0.61, 95% CI 0.29–1.32) and γ-tocopherol (MVRR _tertile3vs1_ = 1.30, 95% CI 0.63–2.69) that became stronger in 4-year lagged analyses. Further investigation is warranted on a potential association between α- and γ-tocopherol and glioma risk.

## Introduction

Gliomas are the most common malignant brain tumors in adults and arise from glial tissue^1^. Patients with these tumors generally have poor prognoses and survival rates^2^. In particular, glioblastoma (GBM), which accounts for more than half of all gliomas (~ 60%), has a 5-year relative survival of approximately 7%^2^.

The brain is comprised of a high content of polyunsaturated fatty acids (PUFAs) with lipids making up 50% of the total mass of neural membranes^3, 4^. Fat-soluble vitamins including vitamins D, A, and E, have been linked with other cancers^5–7^ and could play a role in the etiology of glioma. Vitamin D has receptors throughout the brain and has demonstrated anti-inflammatory and immunomodulation roles^8^. Circulating 25-hydroxyvitamin D (25(OH)D), the accepted measure of vitamin D status, captures exposure to all sources of vitamin D, including sunlight, diet, and supplements^9, 10^. Vitamin A (retinol) participates in numerous, diverse biological events to maintain tissue homeostasis^11^ and has been shown to inhibit the proliferation and migration of cells in primary cultures of human glioma^12^. Vitamin E (α- and γ-tocopherol) plays a pivotal role in preventing lipid peroxidation in brain cell membranes^13^, suggesting potential for a chemo-preventive role in glioma.

In the current study, we examined pre-diagnostic circulating concentrations of 25(OH)D, retinol, and α- and γ-tocopherol in relation to glioma development in three cohorts—UK Biobank (UKB), Nurses’ Health Study (NHS), and Health Professionals Follow-Up Study (HPFS).

## Materials and methods

### Study population and design

#### UK Biobank

UKB is a population-based cohort of 502,536 participants living in the United Kingdom (UK), who were aged 40–69 years at recruitment in 2006 to 2010. Participants were identified from National Health Service patient registries and completed questionnaires that included information on demographics, lifestyle, diet, and health and medical history/conditions. At study enrollment, the majority of participants also provided a 45 mL blood sample. Blood samples were collected and transported overnight at 4 °C to a central laboratory, where they were processed and stored as aliquots at − 80 °C^14^ until analyzed. Participants provided written consent at recruitment, and data were downloaded from UK Biobank Resource under approved protocol 16944.

#### Nurses’ Health Study and Health Professionals Follow-Up Study

NHS, established in 1976, included 121,701 female nurses aged 30–55 years^15^. HPFS began in 1986 and enrolled 51,529 male health professionals aged 40–75 years^16^. All participants completed baseline questionnaires on demographics, lifestyle factors, and medical and other health-related information. Both cohorts have been followed up biennially, with follow-up compliance exceeding 90%^17^. Blood samples were returned by 32,826 NHS participants from 1989–1990 and by 18,018 HPFS participants from 1993 to 1995, through overnight mail. On arrival at a centralized laboratory, the samples were centrifuged to separate plasma from the buffy coat and red cells and were stored in liquid nitrogen freezers until analyzed. Over 95 percent of samples arrived within 26 h of phlebotomy^18, 19^. Among participants with available blood samples, two controls were randomly selected for each glioma case via incidence-density sampling among participants who were still alive and free of cancer at the date of the case diagnosis^20^. Controls were individually matched to the cases on fasting status (fasting vs. non-fasting), year of birth (± 1 year; 2 years was used for 10 matched sets), month of blood collection (± 1 month), and race (white vs. non-white).

The study protocol was approved by the Institutional Review Boards of the Brigham and Women’s Hospital and Harvard T.H. Chan School of Public Health, as well as participating registries (as required).

### Identification of glioma cases

In UKB, participants diagnosed with cancer were identified through linkage to the UK National Health Service Central Register. Glioma cases were those with primary intracranial gliomas (ICD10: C71) and included both GBMs (9440–9441) and lower grade glioma subtypes (non-GBM) (9380–9382, 9400–9401, 9411, 9442, 9450–9451).

In NHS and HPFS, glioma cases were identified initially through self-report with additional cases confirmed by vital status or medical review post-death. Written consent for medical record review was collected from participants or next of kin. Data on tumor subtype (GBM and non-GBM as described above for UKB) was extracted directly from medical records for all cases.

### Laboratory assays

In UKB, serum circulating 25(OH)D was measured in all baseline blood samples by UKB investigators using the DiaSorin Liaison XL immunoassay (http://biobank.ndph.ox.ac.uk/showcase/showcase/docs/serum_biochemistry.pdf). The coefficients of variation (CV) for 25(OH)D among high, medium and low sample quality control concentrations were 5%, 5% and 6%, respectively. Data for retinol (vitamin A) and α- and γ-tocopherol (vitamin E) were not available in UKB.

In NHS and HPFS, plasma circulating 25(OH)D_2_ and 25(OH)D_3,_ retinol, and α- and γ-tocopherol were measured by liquid chromatography-tandem mass spectrometry at Bevital AS (http://bevital.no, Bergen, Norway)^21^. We calculated total plasma circulating 25(OH)D by summing plasma 25(OH)D_2_ and 25(OH)D_3._ Based on blinded quality control samples, in NHS and HPFS, mean intra-assay CVs were 7%, 6% for 25(OH)D, 4%, 4% for retinol, 10%, 10% for α-tocopherol, and 9%, 6% for γ-tocopherol, respectively. As retinol and α- and γ-tocopherol concentrations are correlated with total cholesterol^22^, we considered associations with adjustment for total cholesterol concentrations, which were directly measured by the investigators using an enzymatic assay (Thermo Scientific, Waltham, Massachusetts, United States). CVs for cholesterol were 11% in NHS and 4% in HPFS.

### Statistical analysis

Statistical analyses were performed independently for UKB and NHS/HPFS, as different study designs were implemented and only 25(OH)D was measured in all three studies. In addition, 25(OH)D was measured using different methodologies in UKB compared to NHS and HPFS, and 25(OH)D concentrations have been shown to differ across assay methods^23^. In UKB, we excluded participants with a history of cancer at baseline, those genetically related, and those without a 25(OH)D measurement, leaving 346,812 participants and 444 incident glioma cases in the final analysis. In NHS and HPFS, we included 84 cases (52 from NHS and 32 from HPFS) and 168 controls (104 from NHS and 64 from HPFS) in the final analysis.

To adjust for seasonal variation in each study, 25(OH)D was regressed on the week of blood draw in the full cohort in the UKB and the control participants in NHS/HPFS using sine–cosine functions^24^. In each study, a season-standardized value was calculated for each participant by adding their residual (the difference between their observed concentration and predicted concentration from the regression model) to the predicted average 25(OH)D concentration over the entire year from the regression model in that study^24^. The 25(OH)D concentrations were categorized in two ways—tertiles using cutoffs from the full cohort for UKB and from control participants in NHS/HPFS, and cut-points based on the Institutes of Medicine (IOM) guidelines for bone health: < 50 nmol/L (deficiency/insufficiency), 50 to < 75 nmol/L (sufficiency), and ≥ 75 nmol/L (above sufficiency)^25^. Tests for linear trend were obtained by assigning the median value of a category and then modeling those values as a continuous variable.

In UKB, Cox proportional hazards regression was used to calculate multivariable hazard ratios (MVHRs) and 95% confidence intervals (CIs) for the associations between 25(OH)D and glioma risk. Follow-up consisted of the time from enrollment to cancer diagnosis, death, or last linkage to the National Health Service (October 31, 2015), whichever came first. Multivariable models adjusted for the matching factors used in NHS and HPFS, including age (continuous), fasting status (fasting vs. non-fasting), race (white, non-white), month of blood draw (continuous), and sex (men, women), as well as body mass index (BMI, continuous) and smoking status (never, past, current smoker). Analyses were performed for all glioma cases and separately for GBM and non-GBM subtypes. In the GBM analysis, non-GBM cases were censored at the date of diagnosis and vice versa. Analyses were performed using the “SURVIVAL” package in R version 3.5.0 (Vienna, Austria).

NHS and HPFS, unless otherwise specified, were analyzed as a combined dataset using conditional logistic regression to estimate multivariable risk ratios (MVRR) for the associations between 25(OH)D, retinol, and α- and γ-tocopherol and glioma risk. The distribution in the control participants was used to assign tertile cutpoints for each vitamin. In addition to being conditioned on matching factors, multivariable models were additionally adjusted for BMI (continuous) and smoking status (never, past, current smoker) reported on the questionnaire completed prior to blood draw. For retinol and α- and γ-tocopherol, we also adjusted for plasma total cholesterol (continuous). Analyses were performed for all glioma and by subtype. Among the controls, Spearman correlation coefficients (r) were calculated between the four fat-soluble vitamins and cholesterol in the control participants. Analyses were performed using the SAS statistical package, version 9.4 for UNIX (SAS Institute, Cary, NC).

In all three cohorts, sensitivity analyses were conducted by sex, excluding current smokers, and excluding the cases diagnosed within four years of blood collection. All statistical tests were two-sided with P values < 0.05 considered statistically significant. All methods were carried out in accordance with relevant guidelines and regulations.

## Results

In UKB, average follow-up time was 6.7 (SD: 0.9) years; 444 glioma cases (330 GBM and 114 non-GBM) were identfied. In NHS and HPFS combined, 84 glioma cases (54 GBM and 30 non-GBM) cases were diagnosed, on average, 8.6 (SD: 4.3) years after blood collection. The mean (SD) age at blood collection was 55.7 (8.1) years in UKB, and 60.2 (7.9) years in NHS and HPFS. The NHS and HPFS had a higher proportion of multivitamin users than the UKB (38.1% vs. 17.5%). Only a small proportion of participants were current smokers (UKB: 10.2%, NHS and HPFS: 7.1%); the proportion of never smokers was slightly higher in the UKB (56.1%) than NHS and HPFS (52.4%) (Table 1). Irrespective of source cohort, women and men shared similar characteristics except participants in NHS were younger than those in HPFS (Supplementary Table S1). In the UKB, participants who were diagnosed with glioma during follow-up tended to be older at study enrollment and were less likely to be women compared to the overall study population. By design, in the NHS and HPFS, age and sex were comparable in glioma cases and controls. In the UKB, glioma cases were less likely to be never smokers at study enrollment compared to the entire study population. In NHS and HPFS, glioma cases were also less likely to be never smokers than controls.

**Table 1:** Characteristics of participants in the fat-soluble vitamin and risk of glioma analysis in the UK Biobank, Nurses’ Health Study, and Health Professionals Follow-Up Study.

Characteristics	UKB		NHS and HPFS	
	Cases	Cohort	Cases	Controls
N	444	346,785	84	168
Age, years	59.8 (7.0)	55.7 (8.1)	60.1 (7.9)	60.2 (7.9)
Women, %	37.8	52.8	61.9	61.9
Time to diagnosis, years	4.0 (2.2)	–	8.6 (4.3)	–
Body mass index, kg/m^2^	27.4 (4.4)	27.4 (4.8)	25.0 (4.9)	26.1 (4.6)
Current multivitamin use^1^, %	18.2	17.5	36.9	38.1
Current smoker, %	9.9	10.2	9.5	7.1
Never smoker, %	50.9	56.1	39.3	52.4
25(OH)D^2^, nmol/L	48.5 (19.5)	48.3 (21.0)	71.4 (19.9)	68.3 (20.1)
Retinol, μmol/L	–	–	2.3 (0.6)	2.2 (0.5)
α-tocopherol, μmol/L	–	–	44.0 (19.3)	44.9 (19.4)
γ-tocopherol, μmol/L	–	–	6.3 (3.7)	5.8 (3.0)
Cholesterol, mg/dL	217 (40)	216 (39)	195 (33)	197 (32)
**Season of blood draw (%)**				
Spring	27.0	29.0	23.8	28.6
Summer	22.1	26.5	36.9	33.3
Fall	28.2	24.3	20.2	19.1
Winter	22.7	20.2	19.1	19.1

Continuous variables presented as mean (SD) and categorical variables as percentages (%).

*25(OH)D* 25-hydroxyvitamin D, *HPFS* Health Professionals Follow-Up Study, *NHS* Nurses’ Health Study, *UKB* United Kingdom Biobank.

–Denotes not applicable or data not available.

^1^UKB multivitamin use prevalence includes participants who reported multivitamin or mineral use.

^2^Season-standardized 25(OH)D.

### 25(OH)D

Mean (SD) season-standardized 25(OH)D concentrations were 48.3 (21.0) nmol/L for the UKB cohort and 68.3 (20.1) nmol/L for the controls in the NHS and HPFS studies combined (note: the 25(OH)D concentrations were assessed using different assays and may yield non-comparable results^26^). For UKB, participants in the highest compared with the lowest tertile of 25(OH)D had a nonsignificant 13% lower risk of glioma (MVHR = 0.87, 95% CI 0.68–1.11) and 6% lower risk of GBM (MVHR = 0.94, 95% CI 0.71–1.25). In NHS and HPFS, the MVRR for the same comparison was 0.97 (95% CI 0.51–1.85) for glioma (Table 2). Likewise, no significant associations were observed in UKB or NHS/HPFS when 25(OH)D was modeled using categories based on IOM guidelines for bone health or continuously (Table 2). Results were similar for women and men (Supplementary Table S2), and after excluding current smokers or the cases diagnosed within four years of blood collection (Supplementary Table S3).

**Table 2:** Associations between circulating 25(OH)D and glioma risk in the UK Biobank, Nurses’ Health Study, and Health Professionals Follow-Up Study.

	All gliomas				Glioblastoma (GBM)			
	UKB		NHS and HPFS		UKB		NHS and HPFS	
	Number of glioma cases	HR (95% CI)^1^	Number of glioma cases	RR (95%CI)^2^	Number of GBM cases	HR (95%CI)^1^	Number of GBM cases	RR (95%CI)^2^
**Study specific tertiles**^**3**^								
1	143	1 (ref)	28	1 (ref)	100	1 (ref)	18	1 (ref)
2	150	0.92 (0.73–1.16)	24	0.83 (0.43–1.62)	114	0.99 (0.75–1.31)	15	0.94 (0.41–2.19)
3	151	0.87 (0.68–1.11)	32	0.97 (0.51–1.85)	116	0.94 (0.71–1.25)	21	1.06 (0.48–2.35)
P_trend_^4^		0.25		0.90		0.68		0.90
**IOM guidelines for bone health, nmol/L**								
< 50 (deficiency/insufficiency)	234	0.93 (0.76–1.14)	12	0.80 (0.36–1.74)	170	0.89 (0.70–1.12)	7	0.60 (0.22–1.59)
50 to < 75 (sufficiency)	170	1 (ref)	38	1 (ref)	131	1 (ref)	25	1 (ref)
≥ 75 (above sufficiency)	40	0.72 (0.51–1.02)	34	0.96 (0.52–1.75)	29	0.68 (0.45–1.02)	22	0.88 (0.42–1.85)
P_trend_^4^		0.43		0.74		0.59		0.60
Continuous^5^	444	0.95 (0.85–1.07)	84	1.10 (0.79–1.54)	330	0.96 (0.85–1.10)	54	1.13 (0.76–1.69)

*25(OH)D* 25-hydroxyvitamin D, *CI* confidence interval, *HPFS* Health Professionals Follow-Up Study, *HR* hazard ratio, *IOM* Institute of Medicine, *NHS* Nurses’ Health Study, *RR* risk ratio, *UKB* United Kingdom Biobank.

^1^Adjusted for sex (male, female), age (continuous), race (non-white, white), month of blood collection (continuous), body mass index (continuous), and smoking status (never, past, current).

^2^In addition to conditioning on matching factors in the NHS and HPFS year of birth, fasting status, month of blood collection, and race), adjusted for body mass index (continuous) and smoking status (never, past, current).

^3^Median serum 25(OH)D for each tertile in the UKB: 26.91, 46.43, 68.67 nmol/L; median plasma 25(OH)D for each tertile in the NHS and HPFS: 47.29, 69.49, and 87.07 nmol/L.

^4^The p-value for linear trend corresponds to the p-value of the ordinal variable constructed by assigning the median value of each category to all participants in that category.

^5^Results for the continuous analyses are given for a 25 nmol/L increment.

### Retinol and α- and γ-tocopherol

In NHS and HPFS, among the controls, the mean (SD) concentration was 2.2 (0.5) μmol/L for retinol and 197 (32) mg/dL for cholesterol. We observed a modest correlation among the controls between retinol and total cholesterol (Spearman r: 0.42 in NHS and 0.39 in HPFS) (Supplementary Table S4). Overall, we observed no significant associations for circulating retinol. The MVRRs were 1.16 (95% CI 0.56–2.38) comparing the highest vs lowest tertile and 1.05 (95% CI 0.50–2.21) per SD increment (0.5 μmol/L) in retinol concentration (Table 3). We observed similar results for women and men (Supplementary Table S5), and after excluding current smokers and the matched pairs where the case was diagnosed within four years of blood collection (Supplementary Table S6).

**Table 3:** Association between circulating retinol, α- and γ-tocopherol and glioma risk in the Nurses’ Health Study and Health Professionals Follow-Up Study.

	All glioma		Glioblastoma	
	Number of glioma cases	RR (95% CI)^1^	Number of glioma cases	RR (95% CI)^1^
**Retinol**				
Tertile^2^				
1	24	1 (ref)	15	1 (ref)
2	30	1.10 (0.55–2.21)	22	1.80 (0.75–4.34)
3	30	1.16 (0.56–2.38)	17	1.26 (0.50–3.14)
p_trend_^3^		0.70		0.81
Continuous^4^	84	1.12 (0.85–1.48)	54	1.15 (0.81–1.65)
**α-tocopherol**				
Tertile^5^				
1	34	1 (ref)	22	1 (ref)
2	25	0.71 (0.36–1.39)	16	0.57 (0.24–1.33)
3	25	0.61 (0.29–1.32)	16	0.74 (0.28–1.95)
p_trend_^3^		0.21		0.56
Continuous^4^	84	0.94 (0.68–1.30)	54	1.08 (0.74–1.58)
**γ-tocopherol**				
Tertile^6^				
1	29	1 (ref)	18	1 (ref)
2	26	1.05 (0.53–2.06)	16	0.86 (0.37–2.01)
3	29	1.30 (0.63–2.69)	20	0.94 (0.40–2.25)
p_trend_^3^		0.45		0.93
Continuous^4^	84	1.31 (0.99–1.72)	54	1.06 (0.75–1.50)

*CI* confidence interval, *RR* risk ratio.

^1^In addition to conditioning on matching factors in the NHS and HPFS (year of birth, fasting status, month of blood collection, and race), adjusted for body mass index (continuous), smoking status (never, past, and current), and circulating cholesterol (continuous).

^2^Median plasma retinol for each tertile in the NHS and HPFS: 1.74, 2.16, and 2.74 μmol/L.

^3^The p-value for linear trend across tertiles was the p-value of the ordinal variable constructed by assigning medians to all participants in the tertiles.

^4^Results for continuous analyses for each standard deviation increment.

^5^Median plasma α-tocopherol for each tertile in the NHS and HPFS: 29.66, 41.32, and 57.50 μmol/L.

^6^Median plasma γ-tocopherol for each tertile in the NHS and HPFS: 2.94, 5.60, and 8.59 μmol/L.

In NHS and HPFS, among the controls, the mean (SD) concentration was 44.9 (19.4) μmol/L for α-tocopherol and 5.8 (3.0) μmol/L for γ-tocopherol. For the controls, we observed a stronger correlation between α-tocopherol and total cholesterol (Spearman r: 0.55 in NHS and 0.41 in HPFS) than between γ-tocopherol and total cholesterol (0.24 in NHS and 0.31 in HPFS) (Supplementary Table S4). Compared with the lowest tertile, participants in the highest tertile of α-tocopherol had a 39% lower glioma risk (MVRR = 0.61, 95% CI 0.29–1.32), whereas those in the highest tertile of γ-tocopherol had a 30% elevated glioma risk (MVRR = 1.30, 95% CI 0.63–2.69), although neither association was statistically significant. Results from the continuous analyses generally aligned with the tertile results, with the findings for γ-tocopherol being marginally significant (per SD increment [3.0 μmol/L]: MVRR = 1.31, 95% CI 0.99–2.00) (Table 3). Associations were similar when mutually adjusting α- and γ- tocopherol (data not shown). When cross-classifying by the median concentration of the two tocopherols, we found a 40% reduced risk in the higher α- and lower γ-tocopherol group compared with the lower α- and lower γ-tocopherol group (Supplementary Table S7). Results were similar for women and men (Supplementary Table S5) and after excluding current smokers (Supplementary Table S6). Stronger associations were observed after excluding cases (and their matched controls) diagnosed within the first four years after blood collection (MVRR_tertile 3vs.1_: 0.46 for α-tocopherol and 1.42 for γ-tocopherol) though estimates remained nonsignificant (Supplementary Table S6). Given the association between γ-tocopherol and GBM was different from that for total glioma, we analyzed its association with non-GBM and found a twofold higher risk per SD increment in γ-tocopherol (MVRR = 2.10, 95% CI 1.12–3.93, n = 30 cases).

## Discussion

In three prospective cohorts (UKB from the UK, and NHS and HPFS from the US), statistically significant associations were not observed between circulating 25(OH)D and risk of glioma. In two of these cohorts, NHS and HPFS, associations with retinol and α-and γ-tocopherol were also investigated and were not significantly associated with glioma risk. However, the associations for α-tocopherol and γ-tocopherol were in opposite directions (inverse for α-tocopherol and positive for γ-tocopherol).

Most studies investigating potential roles of fat-soluble vitamins in glioma development have examined associations based on self-reported dietary intake. However, fat-soluble vitamin status in particular^27^, can be affected by numerous factors including bioavailability and specific intrinsic and extrinsic factors, such as food content, health, and genetic characteristics of the population in question^28, 29^. Moreover, dietary assessment of fat-soluble vitamin status can be confounded by other methodological issues. For instance, depending on the time of year, sun-induced vitamin D synthesis in the skin may be another major source of vitamin D. In addition, vitamin E intake typically does not reflect actual levels of various tocopherol isoforms in the body because γ-tocopherol is disproportionately metabolized and excreted^30^. Hence, dietary assessment studies could yield different nutrient-disease associations than studies investigating circulating levels.

There is a paucity of studies investigating vitamin D and glioma risk, and results are inconsistent. In an ecological study, the prevalence of brain cancer was elevated in the southern region of the US^31^. Although this geographical variation could be related to different distributions of genetic and occupational risk factors, it is plausible that geographic differences related to sunlight exposure and vitamin D synthesis could also contribute to the differences in glioma prevalence by region^32^. A nested case–control study from the Janus Serum Bank in Norway^33^ found no overall association between vitamin D status and glioma risk (OR_quintile5vs.1_ = 1.04, 95% CI 0.73, 1.47). Although the authors reported nonsignificant associations in opposite directions in younger (n = 210 cases) and older (n = 185 cases) male participants, the same pattern was not observed in the current study (data not shown). Consistent with a recent Mendelian randomization study that found no evidence for a causal relationship between 25(OH)D and glioma risk^34^, the current analysis, which used directly measured 25(OH)D, also found an overall null association between 25(OH)D and glioma risk.

Vitamin A has been shown to inhibit proliferation and induce differentiation in various cell types mainly through binding to the nuclear retinoic acid receptors (α, β, γ), which are transcriptional and homeostatic regulators whose functions often compromised early in neoplastic transformation^11, 12^. Therefore, vitamin A has been hypothesized to lower risk of glioma. Intake studies of vitamin A have shown inconsistent results. A previous meta-analysis of eight case–control studies suggested a significant inverse association between vitamin A intake and glioma risk^35^. However, there was statistically significant heterogeneity in the study-specific results, as well as the potential for dietary recall and selection bias. A null association between serum retinol and brain cancer risk was observed in the Alpha-Tocopherol, Beta-Carotene Cancer Prevention (ATBC) study (HR_quintile5vs.1_ = 1.03, 95% CI 0.47, 2.25, n = 78 cases)^6^. In NHS and HPFS, we also observed a weak, nonsignificant association for retinol and glioma risk. Failure to identify an association in our study, as well as the ATBC study, may be attributed to small sample size or the tight regulation of circulating retinol^36^.

In the present study, we observed a nonsignificant inverse association between circulating α-tocopherol, but a nonsignificant positive association for γ-tocopherol, with risk of glioma. These findings are in-line with some^37, 38^, but not all prior studies^39^. In a prospective nested case–control study from ATBC Study with 64 glioma cases, circulating α-tocopherol was inversely associated with glioma risk (for a one SD increment, OR 0.65, 95% CI 0.44–0.96); γ-tocopherol was not investigated^38^. Further, a case–control study of 34 GBM cases also reported that prediagnostic α-tocopherol, but not γ-tocopherol, was inversely associated with glioma risk^37^. However, in the Janus Serum Bank study of 110 GBM cases, high α- and γ-tocopherol concentrations were both positively associated with risk of GBM, and the associations were stronger based on samples collected at least 10 years prior to diagnosis^39^. Although we were unable to apply such a long lag due to our limited sample size, we noted a stronger though still nonsignificant positive association for γ-tocopherol in a 4-year lagged analysis, supporting the possibility of a potentially adverse influence of γ-tocopherol on glioma risk. Results from vitamin E dietary intake studies are not consistent with those utilizing biomarkers. A meta-analysis of 12 studies including 3180 glioma cases found that intake of vitamin E from foods or foods and supplements was not associated with risk of glioma^40^. Dietary recall or selection bias issues may have contributed to the different findings, as the meta-analysis mainly included retrospective studies; only one of the studies was prospective in design. Among vitamin E intervention trials^41–52^, only one study has reported results for cancers of the central nervous system with equivalent numbers of cases observed in the treatment (11 cases) and placebo (8 cases) arm; glioma was not reported separately^48^.

Strengths of the current study include the prospective design and availability of pre-diagnostic blood samples, which reduced the possibility of established glioma affecting the measured concentrations of circulating fat-soluble vitamins. In addition, the entire UKB cohort had circulating 25(OH)D measurements, allowing for robust evaluation of potential associations with glioma risk. Although results cannot be directly compared in the UKB and NHS/HPFS studies due to the use of different 25(OH)D assessment methodologies, that could lead to differences in reported 25(OH)D concentrations^26^, we note that neither study supported a role for vitamin D in glioma development. The primary limitations of this study include the small sample size, particularly for the analyses of retinol and α- and γ-tocopherol (84 cases), and use of a single blood sample for each participant. However, in plasma samples collected at two time points 1–2 years apart in NHS, we have shown high reproducibility for 25OH-D (intraclass correlation coefficients [ICC] = 0.71), retinol (ICC = 0.87), and α-tocopherol (ICC = 0.86); γ-tocopherol was not assessed in that study^53^. In addition, the population was mainly Caucasian with limited ethnic diversity. The non-statistically significant inverse association observed for α-tocopherol and non-statistically significant positive association observed for γ-tocopherol require further examination in larger, more ethnically heterogeneous cohorts.

In conclusion, our results suggest circulating 25(OH)D (vitamin D) and retinol (vitamin A) are not associated with glioma risk. Further investigation is however warranted to evaluate the potential associations we observed between vitamin E isoforms, α- and γ-tocopherol, and glioma risk.

## Supplementary Information

Supplementary Information.

## Data Availability

The work is based on the UK Biobank Resource under application number 16944. For the NHS and HPFS cohort data, we provide access through an NIH approved data enclave. Instructions for how to access the data are publicly available through their respective cohort websites.
