# Supplementary material for: Pre-diagnostic circulating concentrations of fat-soluble vitamins and risk of glioma in three cohort studies
Source: Sci Rep. 2021 Apr 29;11:9318. doi: 10.1038/s41598-021-88485-0 (PMC8084971; doi:10.1038/s41598-021-88485-0)
Supplement: Supplementary file 1 — Supplementary Information. [file 41598_2021_88485_MOESM1_ESM.docx]

Pre-diagnostic Circulating Concentrations of Fat-soluble Vitamins and Risk of Glioma in Three Cohort Studies

**Authors:**

Yiyang Yue,^1^ Jordan H. Creed,^2^ David J. Cote,^3,4^ Meir J. Stampfer,^1,3,5^ Molin Wang,^3,5,6^ Øivind Midttun,^7^ Adrian McCann,^7^ Per Magne Ueland,^8,9^ Jeremy Furtado,^1^ Kathleen M. Egan,*^#10^ Stephanie A. Smith-Warner^#1,5^

^#^ Equal contribution as last authors

*denotes corresponding author

**Affiliations:**

^1^ Department of Nutrition, Harvard T.H. Chan School of Public Health, Boston, MA, USA

^2^ Department of Biostatistics and Bioinformatics, H. Lee Moffitt Cancer Center, Tampa, FL, USA

^3^Channing Division of Network Medicine, Brigham and Women’s Hospital, Boston, MA, USA

^4^Computational Neuroscience Outcomes Center, Department of Neurosurgery, Brigham and Women’s Hospital, Boston, MA, USA

^5^Department of Epidemiology, Harvard T.H. Chan School of Public Health, Boston, MA, USA

^6^Department of Biostatistics, Harvard T.H. Chan School of Public Health, Boston, MA, USA

^7^Bevital A/S, Bergen, Norway

^8^Department of Clinical Science, University of Bergen, N‐5021 Bergen, Norway

^9^Laboratory of Clinical Biochemistry, Haukeland University Hospital, Bergen, Norway

^10^Department of Cancer Epidemiology, H. Lee Moffitt Cancer Center, Tampa, FL, USA

**Correspondence:**

Kathleen M. Egan

Department of Cancer Epidemiology, H. Lee Moffitt Cancer Center, Tampa, FL, USA

Kathleen.Egan@moffitt.org

| **Supplementary Table 1.** Characteristics of participants in the fat-soluble vitamins and risk of glioma analysis by sex in the UK Biobank, Nurses’ Health Study, and Health Professionals Follow-up Study. | | | | | | | | |
| --- | --- | --- | --- | --- | --- | --- | --- | --- |
|  | UKB | | | | NHS and HPFS | | | |
| Characteristics | Women | | Men | | Women (NHS) | | Men (HPFS) | |
|  | Cases | Cohort | Cases | Cohort | Cases | Controls | Cases | Controls |
| N | 168 | 182972 | 276 | 163813 | 52 | 104 | 32 | 64 |
| Age, years | 59.8 (6.9) | 55.6 (8.1) | 59.8 (7.0) | 55.8 (8.2) | 57.8 (6.1) | 58.0 (6.1) | 63.8 (9.2) | 63.9 (9.1) |
| Time to diagnosis, years | 4.1 (2.2) | / | 3.9 (2.2) | / | 9.4 (4.3) | / | 7.3 (4.0) | / |
| Body mass index, kg/m^2^ | 27.0 (4.7) | 27.0 (5.2) | 27.6 (4.2) | 27.8 (4.3) | 24.5 (4.9) | 26.6 (5.2) | 25.7 (4.8) | 25.3 (3.4) |
| Current multivitamin use^.^, % | 23.2 | 19.5 | 15.2 | 15.2 | 32.7 | 31.7 | 43.8 | 48.4 |
| Current smoker, % | 7.1 | 8.4 | 11.6 | 12.3 | 13.5 | 10.6 | 3.1 | 1.6 |
| Never smoker, % | 56.0 | 61.1 | 47.8 | 50.5 | 38.4 | 45.2 | 40.6 | 64 |
| 25(OH)D^2^, nmol/L | 48.2 (20.5) | 48.5 (20.9) | 48.6 (19.0) | 48.0 (21.1) | 73.6 (21.0) | 69.2 (22.0) | 67.8 (17.9) | 66.8 (16.7) |
| Retinol, μmol/L | / | / | / | / | 2.3 (0.7) | 2.2 (0.5) | 2.3 (0.4) | 2.3 (0.4) |
| α-tocopherol, μmol/L | / | / | / | / | 47.2 (22.3) | 46.3 (20.4) | 38.7 (11.5) | 42.8 (17.5) |
| γ-tocopherol, μmol/L | / | / | / | / | 7.0 (3.9) | 6.3 (3.0) | 5.1 (2.9) | 5.0 (2.9) |
| Cholesterol, mg/dL | 226 (38) | 221 (38) | 212 (40) | 210 (40) | 201 (36) | 199 (32) | 186 (27) | 194 (33) |
| Season of blood draw, % |  |  |  |  |  |  |  |  |
| Spring | 29.8 | 29.5 | 25.4 | 28.4 | 28.9 | 32.7 | 15.6 | 21.9 |
| Summer | 20.8 | 26.9 | 22.8 | 26.1 | 38.5 | 30.8 | 34.4 | 37.5 |
| Fall | 28.0 | 24.6 | 28.3 | 24.0 | 11.5 | 14.4 | 34.4 | 26.6 |
| Winter | 21.4 | 19.0 | 23.6 | 21.5 | 21.2 | 22.1 | 15.6 | 14.1 |
| Abbreviations: 25(OH)D, 25-hydroxyvitamin D; HPFS, Health Professionals Follow-up Study; NHS, Nurses’ Health Study; UKB, United Kingdom Biobank.  / denotes not applicable or data not available  Continuous variables presented as mean (SD) and categorical variables as percentage (%).  ^1^UKB multivitamin use prevalence includes participants who reported multivitamin or mineral use.  ^2^season-standardized 25(OH)D | | | | | | | | |

| **Supplementary Table 2.** Associations between circulating 25(OH)D and glioma risk by sex in the UK Biobank, Nurses’ Health Study, and Health Professionals Follow-up Study. | | | | | | | | |
| --- | --- | --- | --- | --- | --- | --- | --- | --- |
|  | UKB | | | | NHS and HPFS | | | |
|  | Women | | Men | | Women (NHS) | | Men (HPFS) | |
| Study specific tertiles^3^ | Number of glioma cases | HR (95% CI)^1^ | Number of glioma cases | HR (95% CI)^1^ | Number of glioma cases | RR (95% CI)^2^ | Number of glioma cases | RR (95% CI)^2^ |
| 1 | 54 | 1 (ref) | 89 | 1 (ref) | 15 | 1 (ref) | 13 | 1 (ref) |
| 2 | 55 | 0.92 (0.63-1.136) | 95 | 0.92 (0.68-1.24) | 14 | 0.99 (0.40, 2.41) | 10 | 0.67 (0.24, 1.87) |
| 3 | 59 | 0.94 (0.64-1.39) | 92 | 0.83 (0.61-1.13) | 23 | 1.24 (0.54, 2.84) | 9 | 0.61 (0.19, 1.94) |
| P_trend_^4^ |  | 0.77 |  | 0.23 |  | 0.60 |  | 0.36 |
| IOM guidelines for bone health, nmol/L |  |  |  |  |  |  |  |  |
| <50 (deficiency/insufficiency) | 62 | 0.96 (0.69-1.34) | 108 | 0.91 (0.70-1.18) | 7 | 0.97 (0.34, 2.77) | 5 | 0.75 (0.22, 2.60) |
| 50-<75 (sufficiency) | 91 | 1 (ref) | 143 | 1 (ref) | 21 | 1 (ref) | 17 | 1 (ref) |
| ≥75 (above sufficiency) | 15 | 0.74 (0.42-1.30) | 25 | 0.71 (0.46-1.10) | 24 | 1.20 (0.57, 2.53) | 10 | 0.62 (0.19, 2.04) |
| P_trend_^4^ |  | 0.55 |  | 0.61 |  | 0.61 |  | 0.75 |
| Continuous^5 6^ | 168 | 0.99 (0.83-1.19) | 276 | 0.93 (0.80-1.07) | 52 | 1.13 (0.76, 1.67) | 32 | 1.03 (0.53, 2.00) |
| Abbreviations: 25(OH)D, 25-hydroxyvitamin D; CI, confidence interval; HPFS, Health Professionals Follow-up Study; HR, hazard ratio; IOM, Institute of Medicine; NHS, Nurses’ Health Study; RR, risk ratio; UKB, United Kingdom Biobank.  ^1^Adjusted for sex (male vs female), age (continuous), race (non-white vs white), month of blood collection (continuous), BMI (continuous), and smoking status (never, current and previous).  ^2^In addition to conditioning on matching factors in the NHS and HPFS (age, fasting status, month of blood collection, and race), adjusted for body mass index (continuous) and smoking status (never, current and previous).  ^3^Median serum 25(OH)D for each tertile in the UKB 26.91, 46.43, 68.67 nmol/L; median plasma 25(OH)D for each tertile in the NHS and HPFS: 47.29, 69.49, and 87.07 nmol/L.  ^4^The p-value for linear trend corresponds to the p-value of the ordinal variable constructed by assigning the median value of each category to all participants in that category.  ^5^Results for the continuous analyses are given for a 25 nmol/L increment.  ^6^P for interaction by sex: 0.62 for UKB; 0.81 for NHS and HPFS. | | | | | | | | |

| **Supplementary Table 3.** Associations between circulating 25(OH)D and glioma risk among non-smokers and after a 4-year lag in the UK Biobank, Nurses’ Health Study, and Health Professionals Follow-up Study. | | | | | | | | |
| --- | --- | --- | --- | --- | --- | --- | --- | --- |
|  | Non-smokers | | | | After applying a 4-year lag | | | |
|  | UKB | | NHS and HPFS | | UKB | | NHS and HPFS | |
|  | Number of glioma cases | HR (95%CI)**^1^** | Number of glioma cases | RR (95%CI)**^2^** | Number of glioma cases | HR (95%CI)**^1^** | Number of glioma cases | RR (95%CI)**^2^** |
| Study specific tertiles^3^ |  |  |  |  |  |  |  |  |
| 1 | 118 | 1 (ref) | 25 | 1 (ref) | 70 | 1 (ref) | 21 | 1 (ref) |
| 2 | 139 | 0.97 (0.75-1.25) | 24 | 0.85 (0.42-1.72) | 76 | 0.95 (0.68-1.33) | 21 | 1.02 (0.48-2.15) |
| 3 | 138 | 0.88 (0.68-1.15) | 27 | 1.00 (0.50-2.03) | 75 | 0.91 (0.65-1.29) | 25 | 1.02 (0.48- 2.16) |
| P_trend_^4^ |  | 0.34 |  | 0.98 |  | 0.60 |  | 0.99 |
| IOM guidelines for bone health, nmol/L |  |  |  |  |  |  |  |  |
| <50(deficiency/insufficiency) | 158 | 0.92 (0.74-1.14) | 11 | 0.78 (0.22-2.74) | 84 | 0.90 (0.68-1.21) | 9 | 0.41 (0.09-1.84) |
| 50-<75(sufficiency) | 202 | 1 (ref) | 36 | 1 (ref) | 115 | 1 (ref) | 33 | 1 (ref) |
| ≥75 (beyond sufficiency) | 35 | 0.68 (0.47-0.98) | 29 | 0.75 (0.23-2.45) | 22 | 0.82 (0.51-1.32) | 25 | 0.52 (0.12-2.21) |
| P_trend_^4^ |  | 0.38 |  | 0.79 |  | 1.00 |  | 0.86 |
| Continuous^5 6^ | 395 | 0.94 (0.84-1.07) | 76 | 1.07 (0.74-1.54) | 221 | 0.97 (0.82-1.14) | 67 | 1.08 (0.73-1.58) |
| Abbreviations: 25(OH)D, 25-hydroxyvitamin D; CI, confidence interval; HPFS, Health Professionals Follow-up Study; HR, hazard ratio; IOM, Institute of Medicine; NHS, Nurses’ Health Study; RR, risk ratio; UKB, United Kingdom Biobank.  ^1^Adjusted for sex (male vs female), age (continuous), race (non-white vs white), month of blood collection (continuous), body mass index (continuous), and smoking status (never, current and previous).  ^2^In addition to conditioning on matching factors in the NHS and HPFS (age, fasting status, month of blood collection, and race), adjusted for body mass index (continuous) and smoking status (never, past, and current).  ^3^Median serum 25(OH)D for each tertile in the UKB 26.91, 46.43, 68.67 nmol/L; median plasma 25(OH)D for each tertile in the NHS and HPFS: 47.29, 69.49, and 87.07 nmol/L.  ^4^The p-value for linear trend corresponds to the p-value of the ordinal variable constructed by assigning the median value of each category to all participants in that category.  ^5^Results for the continuous analyses are given for a 25 nmol/L increment. | | | | | | | | |

| **Supplementary Table 4.** Spearman correlation coefficients for fat soluble vitamins and cholesterol concentrations in the Nurses’ Health Study (NHS) and Health Professionals Follow-up Study (HPFS). | | | | | | |
| --- | --- | --- | --- | --- | --- | --- |
|  | | 25 (OH)D | Retinol | α-tocopherol | γ-tocopherol | Cholesterol |
| NHS (Women) *n*=104 | |  |  |  |  |  |
|  | 25(OH)D | 1 | 0.22* | 0.30* | -0.21* | 0.01 |
|  | Retinol |  | 1 | 0.55* | 0.02 | 0.42* |
|  | α-tocopherol |  |  | 1 | -0.15 | 0.55* |
|  | γ-tocopherol |  |  |  | 1 | 0.24* |
|  | Cholesterol |  |  |  |  | 1 |
|  | |  |  |  |  |  |
| HPFS (Men) *n*=64 | |  |  |  |  |  |
|  | 25(OH)D | 1 | 0.11 | 0.10` | -0.18 | -0.04 |
|  | Retinol |  | 1 | 0.32* | 0.12 | 0.39* |
|  | α-tocopherol |  |  | 1 | -0.27* | 0.41* |
|  | γ-tocopherol |  |  |  | 1 | 0.31* |
|  | Cholesterol |  |  |  |  | 1.0 |
| Abbreviations: 25(OH)D, 25-hydroxyvitamin D; HPFS, Health Professionals Follow-up Study; NHS, Nurses’ Health Study; *p<0.05 | | | | | | |

| **Supplementary Table 5.** Associations between circulating retinol, α- and γ-tocopherol, and glioma risk by sex in the Nurses’ Health Study and Health Professionals Follow-up Study. | | | | | |
| --- | --- | --- | --- | --- | --- |
|  |  | Women | | Men | |
|  |  | Number of glioma cases | RR (95%CI)^1^ | Number of glioma cases | RR (95%CI)^1^ |
| Retinol | |  |  |  |  |
|  | Tertile^2^ |  |  |  |  |
|  | 1 | 17 | 1 | 7 | 1 |
|  | 2 | 15 | 1.23 (0.49-3.06) | 15 | 1.09 (0.33-3.54) |
|  | 3 | 20 | 1.14 (0.47-2.77) | 10 | 1.31 (0.37-4.60) |
|  | p _trend_^3^ |  | 0.80 |  | 0.66 |
|  | Continuous^4 5^ | 52 | 1.00 (0.70-1.45) | 32 | 0.76 (0.37-1.56) |
|  | | |  |  |  |
| α-tocopherol | | |  |  |  |
|  | Tertile^6^ |  |  |  |  |
|  | 1 | 18 | 1 | 16 | 1 |
|  | 2 | 14 | 0.48 (0.18-1.27) | 11 | 1.21 (0.44-3.29) |
|  | 3 | 20 | 0.66 (0.23-1.91) | 5 | 0.39 (0.10-1.52) |
|  | p _trend_^3^ |  | 0.55 |  | 0.24 |
|  | Continuous^4 7^ | 52 | 1.39 (0.99-1.95) | 32 | 1.18 (0.68-2.04) |
|  |  |  |  |  |  |
| γ-tocopherol | | |  |  |  |
|  | Tertile^8^ |  |  |  |  |
|  | 1 | 12 | 1 | 17 | 1 |
|  | 2 | 19 | 1.23 (0.48-3.14) | 7 | 0.82 (0.28-2.38) |
|  | 3 | 21 | 1.37 (0.51-3.65) | 8 | 1.28 (0.39-4.17) |
|  | p _trend_^3^ |  | 0.54 |  | 0.76 |
|  | Continuous^4 9^ | 52 | 1.03 (0.74-1.42) | 32 | 1.50 (0.80-2.81) |
| Abbreviations: CI, confidence interval; RR, risk ratio.  ^1^In addition to conditioning on matching factors in the NHS and HPFS (age, fasting status, month of blood collection, and race), adjusted for body mass index (continuous), smoking status (never, past, and current), and circulating cholesterol (continuous).  ^2^Median plasma retinol for each tertile in the NHS and HPFS: 1.74, 2.16, and 2.74 μmol/L.  ^3^The p-value for linear trend across tertiles was the p-value of the ordinal variable constructed by assigning medians to all participants in the tertiles.  ^4^Results for continuous analyses for each standard deviation increment.  ^5^P for ineraction by sex: 0.29 for retinol.  ^6^Median plasma α-tocopherol for each tertile in the NHS and HPFS: 29.66, 41.32, and 57.50 μmol/L.  ^7^P for ineraction by sex: 0.50 for α-tocopherol.  ^8^Median plasma γ-tocopherol for each tertile in the NHS and HPFS: 2.94, 5.60, and 8.59 μmol/L.  ^9^P for ineraction by sex: 0.62 for γ-tocopherol. | | | | | |

| **Supplementary Table 6.** Associations between circulating retinol, α- and γ-tocopherol and glioma risk among non-smokers and after a 4-year lag in the Nurses’ Health Study, and Health Professionals Follow-up Study. | | | | | |
| --- | --- | --- | --- | --- | --- |
|  |  | Non-smokers | | After applying a 4-year lag | |
|  |  | Number of glioma cases | RR (95%CI) | Number of glioma cases | RR (95%CI) |
| Retinol | |  |  |  |  |
|  | Tertile^2^ |  |  |  |  |
|  | 1 | 22 | 1 (ref) | 18 | 1 (ref) |
|  | 2 | 28 | 1.05 (0.51-2.19) | 26 | 1.24 (0.57-2.71) |
|  | 3 | 26 | 1.23 (0.56-2.70) | 23 | 1.17 (0.49-2.80) |
|  | p _trend_^3^ |  | 0.59 |  | 0.75 |
|  | Continuous^4^ | 76 | 1.15 (0.83-1.60) | 67 | 1.06 (0.77-1.46) |
|  |  |  |  |  |  |
| α-tocopherol | | |  |  |  |
|  | Tertile^5^ |  |  |  |  |
|  | 1 | 33 | 1 (ref) | 29 | 1 |
|  | 2 | 23 | 0.68 (0.34-1.37) | 21 | 0.60 (0.28-1.31) |
|  | 3 | 20 | 0.56 (0.24-1.29) | 17 | 0.46 (0.19-1.12) |
|  | p _trend_^3^ |  | 0.17 |  | 0.09 |
|  | Continuous^4^ | 76 | 0.97 (0.66-1.41) | 67 | 0.86 (0.60-1.23) |
|  |  |  |  |  |  |
| γ-tocopherol | | |  |  |  |
|  | Tertile^6^ |  |  |  |  |
|  | 1 | 28 | 1 (ref) | 22 | 1 (ref) |
|  | 2 | 24 | 1.18 (0.58-2.40) | 22 | 1.12 (0.52-2.41) |
|  | 3 | 24 | 1.20 (0.56, 2.60) | 23 | 1.42 (0.62-3.21) |
|  | p _trend_^3^ |  | 0.65 |  | 0.39 |
|  | Continuous^4^ | 76 | 1.32 (0.97-1.79) | 67 | 1.30 (0.96-1.77) |
| Abbreviations: CI, confidence interval; RR, risk ratio.  ^1^In addition to conditioning on matching factors in the NHS and HPFS (age, fasting status, month of blood collection, and race), adjusted for body mass index (continuous), smoking status (never, past, and current), and circulating cholesterol (continuous).  ^2^Median plasma retinol for each tertile in the NHS and HPFS: 1.74, 2.16, and 2.74 μmol/L.  ^3^The p-value for linear trend across tertiles was the p-value of the ordinal variable constructed by assigning medians to all participants in the tertiles.  ^4^Results for continuous analyses for each standard deviation increment.  ^5^Median plasma α-tocopherol for each tertile in the NHS and HPFS: 29.66, 41.32, and 57.50 μmol/L.  ^6^Median plasma γ -tocopherol for each tertile in the NHS and HPFS: 2.94, 5.60, and 8.59 μmol/L. | | | | | |

| **Supplementary Table 7.** Joint associations between plasma α- and γ-tocopherol and glioma risk in the Nurses’ Health Study and Health Professionals Follow-up Study. | | | | | |
| --- | --- | --- | --- | --- | --- |
|  | Lower^1^ γ-tocopherol | | Higher^2^  γ-tocopherol | |  |
|  | Gliomas | RR (95%CI) ^3^ | Gliomas | RR (95%CI) ^3^ |  |
| Lower^1^ α-tocopherol | 22 | 1 (ref) | 25 | 1.06 (0.48-2.34) |  |
| Higher^2^ α-tocopherol | 18 | 0.60 (0.26-1.41) | 19 | 0.84 (0.33-2.14) |  |
| Abbreviations: CI, confidence interval; RR, risk ratio.  ^1^ Below median level.  ^2^Above median level.  ^3^In addition to conditioning on matching factors in the NHS and HPFS (age, fasting status, month of blood collection, and race), adjusted for body mass index (continuous), smoking status (never, past, current), and circulating cholesterol (continuous). | | | | |  |
